# Development of the Physical Activity Research Opportunities (PARO) framework

**DOI:** 10.1186/s12966-025-01843-3

**Published:** 2025-11-26

**Authors:** Laura E. Balis, Daniel P. Hatfield, Meher Din, Sueny Paloma Lima-dos-Santos, Grace Stott, Amanda Sharfman, David R. Brown

**Affiliations:** 1 Center for Nutrition & Health Impact, Omaha, USA; 2 FHI 360, Durham, USA; 3 Graduate College, Ohio University, Athens, USA; 4 Atlanta, USA

**Keywords:** Physical activity, Policy, Practice, Health equity, Translational research, Framework

## Abstract

**Purpose:**

Physical activity is beneficial across the lifespan, but most Americans do not meet physical activity guidelines. Multiple sources exist that identify opportunities to address gaps in physical activity research knowledge and implementation. Several of these opportunities have important considerations for populations experiencing health inequities. The goal of this study was to identify, synthesize, and categorize opportunities for research (i.e., systematic investigations to develop generalizable knowledge) into a conceptual framework to advance physical activity research in a cohesive and efficient manner.

**Methods:**

The National Collaborative on Childhood Obesity Research convened subject matter experts to conduct five qualitative document analysis steps: (1) identify sources published by United States government, intergovernmental, or national non-profit organizations related to physical activity, (2) review sources to extract research opportunities, (3) code the opportunities by variables (translational research phase, social ecological level, setting, and priority population) determined by the expert group, (4) synthesize data on similar opportunities, and (5) review crosstabulation data to examine coding patterns and develop a framework.

**Results:**

Opportunities (*n* = 385) were extracted from sources (*n* = 11) and combined into condensed opportunity statements (*n* = 87). Most called for effectiveness research (*n* = 44, 51%) or dissemination and implementation science research (*n* = 14, 16%). 38% were related to policy, systems, and environmental interventions (*n* = 33), and 70% mentioned community settings (*n* = 61). Additionally, 76% did not include health equity considerations (*n* = 66), and 75% mentioned no specific population or populations across the lifespan (*n* = 65). The resultant Physical Activity Research Opportunities (PARO) framework details opportunities by translational research phase (methods/measures development, etiology, efficacy, effectiveness, dissemination and implementation, and surveillance) and social ecological level (individual or interpersonal, policy/systems/environmental, and crosscutting), including health equity considerations.

**Conclusions:**

The resultant PARO framework highlights gaps in current evidence and reveals opportunities for physical activity funders, researchers, policymakers, and practitioners to strategically advance their work. There are prospects for designing efficacy and effectiveness trials with an eye toward dissemination and implementation, developing strategies for improving dissemination and implementation, and using community- and practitioner-engaged approaches across translational research phases to advance health equity. Health equity can also be addressed by tailoring interventions, enhancing reach to priority populations, and improving social determinants of health.

**Supplementary Information:**

The online version contains supplementary material available at 10.1186/s12966-025-01843-3.

## Background

Physical activity research emerged in the 1950 s with an initial focus on epidemiology and health outcomes, which was followed by research on determinants and correlates, policies, and, more recently, intervention studies [1]. There has been much growth in the field, and it is well recognized that regular physical activity prevents, delays the onset of, and helps with improvement and management of chronic diseases. Physical activity also contributes to extended life expectancy and increased resilience (i.e., the ability to respond to stressors) [2–6]. Recent evidence also demonstrates the benefits of physical activity for reducing the severity of infectious diseases [7]. Evidence for optimal types and doses (i.e., frequencies, intensities, and durations) of physical activity have been established for many health outcomes, and multiple countries have published physical activity guidelines [8–11].

In the United States, two editions (2008 and 2018) of the Physical Activity Guidelines for Americans, and corresponding midcourse reports, have been published [12–15]. Although the national guidelines provide recommended levels of moderate-to-vigorous intensity aerobic physical activity and muscle-strengthening activity for adults, and in spite of multiple compilations of evidence-based interventions that increase physical activity levels [16–18], the majority of adult Americans (76.5%) self-report failing to meet these guidelines [19]. Progress toward Healthy People 2030 goals of increasing the proportion of Americans meeting guideline recommendations is slow [20], and there are disparities in physical activity levels across race, ethnicity, gender, education level, age, and geographic location [21, 22].

This gap between research (evidence-based recommendations) and practice (meeting the recommendations) is not unique to physical activity. Across prevention science and health care disciplines, the translation of empirical research findings to real-world applications can take 15–17 years [23–26]. Dissemination and implementation science is a discipline dedicated to accelerating the translation of research to practice [27, 28]. Dissemination and implementation science researchers use specific methods, models, and measures to understand contextual factors that influence intervention adoption, implementation, and sustainability [29, 30]; test implementation strategies (methods or techniques of moving research to practice); and measure implementation outcomes (how much and how well evidence-based interventions are implemented) [31–35]. Beyond the challenges in real-world practice settings, other reasons for the research-to-practice time lag include the traditional research pipeline (i.e., translational phases that move from efficacy to effectiveness to dissemination and implementation); academic publication requirements; and funder priorities, which can affect the types of research conducted and available for dissemination to and implementation in practice settings [31, 36].

For example, the majority of physical activity research in the last two decades has been observational or descriptive studies focused on prevalence or health consequences, and there have been calls for more studies testing intervention effectiveness or dissemination and implementation [1, 36, 37]. Of the intervention studies conducted over the last two decades, most have tested individual- or interpersonal-level interventions [38], although research conducted since the early 2000 s has emphasized the importance of policy, systems, and environmental (PSE) and multi-level interventions [1, 39]. It is recognized that individual-level interventions alone are not sufficient, and supportive environments are needed to improve adherence to physical activity recommendations [40–43]. Lastly, an additional challenge to the gap between physical activity science and practice is that research often addresses familiar hypotheses rather than asking and answering novel questions that could improve health [1].

Focusing the physical activity research agenda is important to informing studies to address gaps across the translational research spectrum (i.e., inquiries that move scientific observations into implementable health-promoting interventions) [44], answer priority public health questions, and support the scale-up of effective interventions in diverse communities across the United States [36]. Government, intergovernmental, and national non-profit organizations have begun this work by publishing research opportunities, knowledge gaps, and evidence gaps (herein called opportunities) in consensus documents, guidelines, reports, and other sources [8, 10, 45–52]. Sources have called for better surveillance, additional policy research, and more studies conducted in real-world settings (or contexts) [8, 10, 45–52]. However, this information is housed in disparate sources, which can be challenging for researchers, practitioners, funders, policy makers, and other interested users to access and navigate.

Launched in 2009 to accelerate progress in reducing childhood obesity, the National Collaborative on Childhood Obesity Research (NCCOR) is a public-private partnership of four leading childhood obesity research funders: the Centers for Disease Control and Prevention (CDC), National Institutes of Health (NIH), Robert Wood Johnson Foundation, and U.S. Department of Agriculture [53]. One of NCCOR’s goals is to provide national leadership to advance implementation of evidence-based interventions, including those addressing physical activity [53]. Consistent with this goal, subject matter experts at the CDC and NIH sought to identify and organize physical activity research opportunities from disparate sources into one conceptual framework to advance physical activity promotion research agendas [54].

A conceptual framework of physical activity research opportunities is likely to serve several functions. First, a framework can enhance communication of research opportunities and maximize overall research efficiency. Second, a framework can provide the research community, funding agencies, and policy makers with a tool to identify priority areas and topics in physical activity and build cross-sector collaborations. Third, a framework can guide the strategic planning of future activities, including updates to the Physical Activity Guidelines for Americans [55] that synthesize knowledge on physical activity prevalence and evidence-based interventions. Thus, the objective of this research was to categorize physical activity research opportunities from existing physical activity sources in a single conceptual framework.

## Methods

Qualitative document analysis [56, 57] combined with expert consultation was used, as done in other framework development studies [58, 59]. CDC and NIH subject matter experts on the NCCOR project team were consulted throughout the five-step qualitative document analysis process, which consisted of (1) *source identification*: identifying sources describing opportunities for physical activity research, (2) *source review*: reviewing sources to extract opportunities (i.e., research opportunities, knowledge gaps, and evidence gaps relevant to advancing physical activity research), (3) *source coding*: coding the opportunities by variables (i.e., categories to be analyzed) determined by the expert group, (4) *data synthesis*: synthesizing data on similar opportunities, and (5) *framework development*: reviewing crosstabulation data to examine relationships between variables and develop the Physical Activity Research Opportunities (PARO) framework.

### Source identification

First, CDC and NIH subject matter experts completed a collaborative scanning exercise to identify sources detailing opportunities for advancing physical activity research. Each expert suggested United States government, intergovernmental (e.g., World Health Organization), and non-profit organization sources based on their relevant experience. Sources needed to be published in 2012 or later to align with the first Physical Activity Guidelines Midcourse Report [14] and focus on the most recent opportunities, knowledge gaps, and evidence gaps, even if related to evidence-based recommendations generated prior to 2012. Sources containing multiple summaries of evidence-based interventions (e.g., the Community Preventive Services Task Force Findings for Physical Activity [17] and U.S. Preventive Services Task Force Recommendation Topics [60]) were considered single sources; each evidence summary related to physical activity was included.

### Source review

Next, the included sources were scanned to identify and extract research opportunities [59]. Seven researchers extracted opportunities into an Excel file. Opportunities were extracted if they were (1) related to physical activity (e.g., recommendations related only to nutrition were not included) and (2) relevant to advancing physical activity research.

Two researchers reviewed the extracted opportunity statements to ensure they included one unique idea, were relevant to physical activity research, and were clear and understandable [61]. Opportunity statements that included multiple concepts were separated into individual statements. For example, the extracted statement “[Communities should] Conduct periodic evaluation of existing community physical activity programs to ensure that all community members have the opportunity to engage in evidence-based/informed physical activity promoting programs, and develop new programs as gaps in coverage are identified.” was split into two statements: “[Communities should] Conduct periodic evaluation of existing community physical activity programs to ensure that all community members have the opportunity to engage in evidence-based/informed physical activity promoting programs” and “[Communities should] develop new [community physical activity] programs as gaps in coverage [community member participation] are identified.” Additional text from sources was added as needed to clarify statements that were unclear. Finally, duplicate statements (e.g., where the same phrase was present multiple times in a source and extracted more than once) were removed.

### Data coding

After reviewing the list of opportunity statements, the expert group discussed variables that could be used to categorize the statements into a framework. A coding guide (Table 1) was developed based on four variables: translational phase, social ecological level, priority population, and setting. The translational phase variable was developed by adapting standard translational research models that detail moving scientific observations into health-promoting interventions [44]. Existing models include phases moving from “bench to bedside” to population-level impacts: basic research, pre-clinical trials, clinical research, clinical implementation, and public health [44, 62]. However, these models were developed for biomedical sciences [62], and the bench science phases were less relevant for understanding physical activity research opportunities. Thus, the model was adapted to include seven relevant phases often included in public health research and practice models: (1) methods/measures development, (2) etiology, (3) intervention development, (4) efficacy, (5) effectiveness, (6) dissemination and implementation, and (7) surveillance [36, 63–65].

**Table 1:** Coding guide for physical activity research opportunities included in the qualitative document analysis

Category	Code	Definition
Translational research phase	Methods/measures development	Research focused on the creation, refinement, and validation of tools and methods for measuring physical activity impacts and implementation and ensuring their reliability and relevance across diverse contexts and populations.
	Etiology	Research that investigates the causes or origin of physical activity patterns and practices, including barriers, facilitators, and determinants.
	Intervention development	Research focused on designing and developing interventions (e.g., programs, practices, policies) targeting improved physical activity outcomes.
	Efficacy	Research that needs to be done in a tightly controlled setting (e.g., through a randomized controlled trial) to understand efficacy of developed interventions under ideal conditions.
	Effectiveness	Research that needs to be conducted in a pragmatic setting to understand effectiveness and public health impacts of developed interventions under real-world conditions (e.g., with few restrictions on participants or delivery agents).
	Dissemination and implementation	Research or practice that intervenes to improve intervention adoption (uptake), implementation (cost or fidelity), maintenance (or sustainability or institutionalization), scaling up or out (to new populations and/or settings), or dissemination.
	Surveillance	Research that monitors and tracks health physical activity rates, policies, and environments over time for public health purposes.
Social ecological level	Individual and interpersonal	Research on interventions that primarily focus on individuals or their immediate social circles, such as family or friends.
	Policy, systems, and environment	Research that targets larger-scale changes, including laws, regulations, and public policies, as well as broader systems or the overall environment, which includes physical factors such as neighborhood design.
	Crosscutting or unspecified	Research that refers to interventions taking place across multiple levels or does not specifically focus on or mention any defined social ecological level.
Priority population	Children or adolescents	Research that involves participants who are children or adolescents (< 18 years old).
	Adults	Research that primarily involves adult populations (18–64 years old).
	Older adults	Research that primarily involves older adults (65 or older).
	Across the lifespan	Research that doesn’t mention a specific priority population or that prioritizes multiple age groups.
Setting	Clinical	Research is conducted in clinical settings, including hospitals, clinics, or other healthcare institutions.
	Community	Studies are conducted in community settings, such as schools, workplaces, or any non-clinical setting of daily life.
	Not specified	Research that does not specify a setting.
Health equity	Yes	Opportunities that mention social determinants of health or health disparities; reach, representativeness, or analysis by demographic items; intervention tailoring to improve reach; or other methods of reaching diverse priority populations.
	No	Opportunities that do not explicitly mention social determinants of health or health disparities; reach, representativeness, or analysis by demographic items; intervention tailoring to improve reach; or other methods of reaching diverse priority populations.

Social ecological level was based on the Social Ecological Model [43, 66] and informed by the Health Impact Pyramid [40]. The models were condensed to classify interventions into two levels (individual and interpersonal; policy, systems, and environment) or as crosscutting interventions or unspecified (i.e., explicitly spanning multiple levels or not specifically focused on a defined social ecological level). The social ecological level categorization refers to the level of influence targeted through an intervention (e.g., whether it intervenes to change individuals’ or families’ knowledge or skills or intervenes to establish policies or modify the environment), not whether the goal is to change behaviors among individuals or groups. For example, active transportation interventions typically work by modifying policies or environments (e.g., through Complete Streets policies [67] or biking/walking paths that connect community destinations [68]) with a goal of increasing individuals’ physical activity levels; active transportation interventions are considered PSE-level interventions.

Priority populations were defined in alignment with the Physical Activity Guidelines for Americans [12] and included children and adolescents (3–17 years), adults (18–64 years), older adults (65 + years), and no specific population or populations across the lifespan. Finally, settings were defined as clinical, community (settings outside of health care that influence individuals’ daily lives) [69], or not specified.

Two researchers coded the first 30 opportunity statements and met to reconcile and refine the coding guide. Next, pairs of researchers independently coded each opportunity and reconciled discrepancies with assistance from the lead researcher as needed.

### Data synthesis

The next stage involved synthesizing the opportunities to group similar concepts into broad overarching statements to be used in the framework. One researcher sorted opportunity statements into multiple combinations of variables (e.g., all opportunity statements that were coded as efficacy research and individual or interpersonal level; all opportunity statements coded as dissemination and implementation research and community settings). Statements within each grouping were reviewed, and those that were similar and could be condensed were noted. Condensed opportunity statements could include original statements that spanned multiple phases but were deemed most similar based on other variables. The lead researcher reviewed and finalized the suggested opportunities to be condensed and grouped together and wrote new statements.

The final step of the synthesis process was coding the condensed opportunity statements. The expert group recommended adding a variable (dichotomous yes/no) for health equity considerations because of the need for equity considerations in physical activity research [22, 70, 71]. Opportunities statements were coded as including health equity considerations if they explicitly mentioned social determinants of health or health disparities; reach, representativeness, or analysis by demographic items; intervention tailoring to improve reach; or other methods of reaching diverse priority populations (Table 1). Two researchers independently coded the condensed opportunity statements into the final five variables (translational phase, social ecological level, priority population, setting, and health equity considerations) and met to reconcile discrepancies through discussion and agreement.

### Framework development

Crosstabulation analysis was completed for each pairwise combination of original coding variables (translational phase, social ecological level, priority population, and setting; six total combinations) to examine relationships between variables. Two researchers reviewed crosstabulation output to determine the ideal combination of variables to organize the condensed opportunity statements into the final framework. Potential combinations were reviewed with the subject matter experts, and a combination of two variables was selected through discussion and consensus.

## Results

Results are presented for each of the five steps of the qualitative document analysis process: *source identification*, *source review*,* data coding*,* data synthesis*, and *framework development.*

### Source identification

Sources (*n* = 11) published between 2012 and 2022 were identified. Most (*n* = 7, 64%) were authored by government or intergovernmental organizations. See Table 2 for a list of each source, including year published, organization, description, and number of statements extracted. Opportunity statements (*n* = 385 after duplicates were removed) were extracted from sources.

**Table 2:** Sources (*n* = 11) and statements (*n* = 385) included in the qualitative document analysis

Source	Year	Organization	Description	Statements extracted *n* (%)
Community Preventive Services Task Force (CPSTF) Findings for Physical Activity [17]	2022	Guide to Community Preventive Services	Contains multiple summaries of evidence-based interventions reviewed by the CPSTF.	104 (27)
2018 Physical Activity Guidelines Advisory Committee Scientific Report [12]	2018	U.S. Department of Health and Human Services	Provides a detailed summary of the disease prevention and health promotion benefits of a more physically active America that is firmly established by the latest scientific evidence.	103 (27)
National Physical Activity Plan [72]	2016	Physical Activity Alliance	A comprehensive set of policies, programs, and initiatives designed to increase physical activity in all segments of the U.S. population.	41 (11)
Step it up! The Surgeon General’s Call to Action to Promote Walking and Walkable Communities [73]	2015	U.S. Department of Health and Human Services	Aims to get Americans walking and wheelchair rolling for the physical activity needed to help prevent and reduce their risk of chronic diseases and premature death.	31 (8)
Physical Activity Guidelines for American-Midcourse Report: Strategies to Increase Physical Activity Among Youth [14]	2012	U.S. Department of Health and Human Services	Intended to identify interventions that can help increase physical activity in youth across a variety of settings.	26 (7)
Educating the Student Body: Taking Physical Activity and Physical Education to School [74]	2013	Institute of Medicine	Makes recommendations about approaches for strengthening and improving programs and policies for physical activity and physical education in the school environment.	24 (6)
Strategic Priorities for Physical Activity Surveillance in the United States [75]	2016	Centers for Disease Control and Prevention and the American College of Sports Medicine	Identifies strategic priorities to develop a national plan for physical activity surveillance similar to the U.S. National Physical Activity Plan [76] for promotion.	19 (5)
Advancing Measurement for High-Risk Populations and Communities Related to Childhood Obesity [77]	2020	National Collaborative on Childhood Obesity Research	Makes recommendations for researchers, practitioners, and funders to address measurement gaps.	18 (5)
Advancing the Global Physical Activity Agenda: Recommendations for Future Research by the 2020 WHO Physical Activity and Sedentary Behavior Guidelines Development Group [47]	2020	World Health Organization	Identifies gaps in the existing literature on the amount of physical activity and sedentary behavior associated with optimal health in specific populations.	12 (3)
U.S. Preventive Services Task Force Recommendation Topics [60]	2022	U.S. Preventive Services Task Force	Systematically reviews the evidence of effectiveness and develops multiple summaries of evidence-based interventions for clinical preventive services.	5 (1)
Biden-Harris Administration National Strategy on Hunger, Nutrition, and Health [78]	2022	White House	Identifies actions the Biden-Harris administration will pursue across five pillars related to nutrition, physical activity, and health.	2 (1)

### Source review

Of the 385 opportunity statements, the majority were extracted from the Community Preventive Services Task Force (CPSTF) Findings for Physical Activity [17] (*n* = 104, 27%) and the 2018 Physical Activity Guidelines Advisory Committee Scientific Report [12] (*n* = 103, 27%). See Supplemental File 1 for the list of opportunity statements by source.

### Data coding

Opportunity statements were primarily related to effectiveness (*n* = 187, 49%) or efficacy (*n* = 65, 17%) research. Most were related to crosscutting interventions (*n* = 147, 38%), unspecified (i.e., community and/or clinical) settings (*n* = 141, 37%), and either no specific population or populations across the lifespan (*n* = 246, 64%).

### Data synthesis

Opportunities were combined into condensed opportunity statements (*n* = 87) that primarily called for effectiveness research (*n* = 44, 51%) or dissemination and implementation science research (*n* = 14, 16%). 38% of the condensed statements were related to PSE interventions (*n* = 33), and 70% mentioned community settings (*n* = 61). Additionally, 75% either mentioned populations across the lifespan or did not specify a life stage (*n* = 65), and 66 statements (76%) did not include health equity considerations. See Supplemental File 2 for the list of condensed opportunity statements, including the number of original statements condensed into each.

### Framework development

Upon review, two of the six variable combinations (social ecological level and setting, priority population and setting) lacked granular data for developing a framework. The remaining four combinations (translational phase and social ecological level, translational phase and priority population, translational phase and setting, social ecological level and priority population) were reviewed by the subject matter experts, and the group deemed translational phase and social ecological level (with health equity considerations highlighted) as best presenting the data. See Supplemental File 3 for crosstabulations of physical activity opportunity statements by translational phase and social ecological level. The PARO framework is presented in Tables 3, 4, 5, 6, 7 and 8, with one table per translational phase. Within each table, condensed physical activity research opportunity statements are organized by social ecological level, and health equity considerations are highlighted. The tables also indicate the number of original statements from which the condensed opportunity statements were derived and note the sources from which the original statements were extracted.

**Table 3:** Methods/measures development: opportunities (*n* = 8) by social ecological level, with health equity considerations

Social ecological level	Methods/measures development opportunities	Health equity*	Source references	Original statements (*n*)
Individual and interpersonal	Develop tools or apps to measure experiences of sports participants.		[72]	3
Policy, systems, and environment	Develop conceptual frameworks and systemic measures of social determinants of health.	x	[77]	2
	Develop analysis to quantify overall impacts (e.g., fuel use, air pollution, economic) of active transportation.		[17, 72, 73, 75]	5
	Develop, test, and refine improved walkability measures.		[17, 73]	5
	Develop standard data collection methods, measures, and models for pedestrian and bicyclist counts.		[73, 74]	4
Crosscutting	Develop, test, and disseminate physical activity behavior measures for specific populations to enable synthesis across studies.	x	[77, 78]	3
	Use community-engaged processes to develop, test, and disseminate multi-level physical activity measures for diverse populations.	x	[72, 73, 75, 77]	7
	Assess reliability and validity of surveillance questions.		[75]	3

* An “x” in the health equity column denotes opportunity statements that mention social determinants of health or health disparities; reach, representativeness, or analysis by demographic items; intervention tailoring to improve reach; or other methods of reaching diverse priority populations

**Table 4:** Etiology: opportunities (*n* = 6) by social ecological level, with health equity considerations

Social ecological level	Etiology opportunities	Health equity*	Source references	Original statements (*n*)
Individual and interpersonal	Study how parent-child interactions and other components of family life influence physical activity levels.		[14, 77]	2
Policy, systems, and environment	Assess the effects of crime, proximity from everyday destinations, and other features of built environment interventions on physical activity patterns.	x	[14, 17]	4
	Investigate proximal outcomes of mass media campaigns (knowledge, intentions, attitudes, beliefs).		[17]	2
	Investigate school built environment features and disparities that influence physical activity.		[74]	2
Crosscutting	Identify populations at risk for physical inactivity and conduct research to understand their specific barriers.	x	[72]	2
	Examine differences in health effects between various types of sedentary time (occupational, class/study, screen time) and postures (sitting, reclining).		[47]	2

*An “x” in the health equity column denotes opportunity statements that mention social determinants of health or health disparities; reach, representativeness, or analysis by demographic items; intervention tailoring to improve reach; or other methods of reaching diverse priority populations

**Table 5:** Efficacy: opportunities (*n* = 6) by social ecological level, with health equity considerations

Social ecological level	Efficacy opportunities	Health equity*	Source references	Original statements (*n*)
Individual and interpersonal	Build primary evidence for postnatal interventions, including among specific populations (racial/ethnic groups, women with gestational diabetes or postnatal depression).	x	[12]	3
	Build evidence of efficacy for primary care physical activity interventions.		[12, 14, 72, 78]	10
	Conduct experimental research to determine associations between physical activity/sedentary time with maternal and fetal outcomes.		[47]	4
	Conduct rigorous experimental studies to understand the effectiveness and individual-level maintenance of sedentary behavior interventions.		[12, 47, 74]	14
Crosscutting	Improve the internal validity of physical activity research, including appropriate comparator arms for efficacy trials.		[12]	2
	Improve the understanding of intermittent versus sustained physical activity on disease risk factors.		[74]	2

* An “x” in the health equity column denotes opportunity statements that mention social determinants of health or health disparities; reach, representativeness, or analysis by demographic items; intervention tailoring to improve reach; or other methods of reaching diverse priority populations

**Table 6:** Effectiveness: opportunities (*n* = 44) by social ecological level, with health equity considerations

**Social ecological level**	**Effectiveness opportunities**	Health equity*	**Source references**	**Original statements** (*n*)
Individual and interpersonal	Evaluate the effectiveness of home-based exercise interventions for older adults on specific populations, including by gender, age group, race/ethnicity, income, and disability status.	x	[12, 17]	9
	Evaluate the impact of individually adapted behavior change programs, including reach, effectiveness (including unintended consequences), cost-effectiveness, and implementation (physical activity type, delivery mode).	x	[12, 17]	8
	Examine the effectiveness of wearable activity monitors by age, race/ethnicity, socioeconomic status, and overweight/obesity.	x	[12, 17]	7
	Examine the reach, effectiveness (including unintended consequences), implementation, and individual-level maintenance of digital health interventions for adults age 55+.	x	[17]	4
	Build evidence for providing rewards and incentives based on achieving physical activity goals.		[12]	7
	Build evidence of effectiveness for interventions taking place in early care and education settings.		[12, 14]	5
	Build evidence on the effectiveness of active video games for youth.		[12]	3
	Build evidence on the effectiveness of social media interventions for adults and youth.		[12]	4
	Build evidence on the effectiveness of text messaging and app-delivered interventions for adults and youth.		[12]	6
	Compare family and home-based intervention effectiveness by demographic characteristics.		[14, 17]	2
	Determine the effectiveness of web-based interventions for adults with type 2 diabetes.		[12]	2
	Determine the effects of home-based exercise interventions for older adults on injuries, morbidity/mortality, falls, mental health outcomes, and quality of life.		[12, 17]	4
	Examine impacts (clinical and health outcomes) of wearables combined with behavior change strategies.		[12, 17, 73, 78]	5
	Examine the effectiveness of computer-tailored interventions, including adverse events and cost-effectiveness.		[12]	6
	Investigate the effectiveness and implementation (specific advice, reason for referral, payment scheme) of physical activity prescriptions.		[12]	2
	Investigate the effectiveness of nurse-delivered interventions in community settings, including by specific physical activity prescription (frequency, intensity, time, type).		[12]	2
	Test specific strategies to promote physical activity for family members of different ages at home.		[12, 14, 17, 72]	5
Policy, systems, and environment	Conduct research to understand effectiveness of mass media campaigns by dose, channel, and priority population.	x	[12, 17, 72]	8
	Determine the economic effects of neighborhood displacement on historically excluded populations as a result of park, trail, and greenway interventions.	x	[17]	2
	Study which interventions or combinations of interventions are most effective in addressing barriers to use of parks, trails, or greenways among specific populations (older adults, people with disabilities, people with lower incomes).	x	[17]	6
	Study the effects of increased access to parks and facilities in communities with less access and in populations experiencing health disparities.	x	[12, 17]	3
	Build evidence on community-wide interventions, including evidence-based core components and reach.		[12, 60, 74]	3
	Build evidence for indoor/outdoor recreation facilities combined with trail/greenway infrastructure in rural settings.		[12, 17]	2
	Conduct more longitudinal studies to capture magnitude of change in physical activity as a result of built environment interventions.		[17]	3
	Conduct natural experiments to evaluate the effects of policy and environment changes on health outcomes.		[12, 14, 72, 73, 77]	9
	Determine the cost-effectiveness of park, trail, and greenway infrastructure interventions (alone and with other interventions).		[17]	2
	Evaluate the effectiveness and overall public health impact of community-wide campaigns, including by delivery setting and channel.		[17, 72, 73]	5
	Evaluate interventions to understand the relationship between built environment improvements, physical activity patterns, and pedestrian/cyclist injuries.		[12, 72, 73]	8
	Examine the effectiveness of park, trail, and greenway infrastructure improvements in improving perceptions of crime and safety.		[17, 72]	3
	Investigate the effectiveness and maintenance of point-of-decision prompts, including periodic boosters.		[17]	3
	Study the combined effects of park/trail/greenway infrastructure improvements with other interventions (e.g., programming, access, community engagement).		[17]	6
Crosscutting	Collaborate with faith-based organizations to deliver and test accessible and tailored programs.	x	[72]	3
	Determine the effectiveness of school-based physical activity interventions by race/ethnicity, socioeconomic status, disabilities, culture, and school location/resources.	x	[12, 17, 74]	9
	Study the reach of community-based physical activity programs and identify gaps.	x	[72]	2
	Analyze the effectiveness of physical activity interventions in school settings, including on academic achievement.		[12, 14, 17, 74]	18
	Assess the economic benefits of active travel to school.		[17]	2
	Build evidence for the effectiveness of sedentary behavior interventions in worksites, including dose-response and adverse events.		[12]	6
	Conduct more rigorous studies of youth physical activity interventions, including adverse effects and cost-effectiveness.		[12, 47]	3
	Determine cost-effectiveness and comparative cost-effectiveness of physical activity interventions.		[12, 14, 73]	6
	Determine specific messages or combinations of messages that are most effective for community-wide physical activity campaigns.		[17, 60, 73]	2
	Determine the effectiveness of active travel to school interventions on physical activity levels.		[12, 17]	3
	Examine the effectiveness and implementation of faith-based programs, including duration and theoretical basis.		[12, 17]	2
	Improve the understanding of the effectiveness of physical activity interventions on motor competence, cognition, and learning in youth.		[74]	3
	Study the implementation of school-based physical activity interventions, including duration, frequency, and type.		[12, 14, 17, 74]	5

*An “x” in the health equity column denotes opportunity statements that mention social determinants of health or health disparities; reach, representativeness, or analysis by demographic items; intervention tailoring to improve reach; or other methods of reaching diverse priority populations

**Table 7:** Dissemination and implementation: opportunities (*n* = 14) by social ecological level, with health equity considerations

Social ecological level	Dissemination and implementation opportunities	Health equity*	Source references	Original statements (*n*)
Individual and interpersonal	Tailor and test existing school-based physical activity interventions for student needs and school context.	x	[17, 72, 74]	4
	Assess the implementation of computer-tailored interventions, including tailoring variables and core components.		[12]	2
	Investigate the implementation of social support interventions in community settings, including type, medium, intensity, structure, and frequency.		[12, 17]	5
Policy, systems, and environment	Conduct policy research on improving the adoption and implementation of active transportation, land use, and community design policies.		[60, 73, 75]	3
	Determine evidence-based core components of active travel to school interventions (e.g., active school buses, school bus availability, proximity to homes).		[14, 17]	2
	Determine which characteristics of park, greenway, and trail infrastructure (e.g., wayfinding signs, trails, fitness centers) are most effective for increasing physical activity.		[17]	3
	Study the implementation of point-of-decision prompts, including format, type, size, distance to stairs, and number of flights.		[17]	3
	Test strategies to integrate physical activity interventions in clinical settings and guidelines.		[72]	4
Crosscutting	Conduct research on the effectiveness of technical assistance to support communities in collecting data, measuring outcomes, and tailoring interventions.	x	[72, 73, 75, 77]	8
	Develop and systematically test methods for improving the implementation and sustainability of evidence-based physical activity interventions in real-world settings.		[12, 14]	2
	Examine how to best integrate and disseminate faith-based and faith-placed interventions.		[72]	3
	Examine barriers to adoption, implementation, and maintenance of school-based physical activity interventions.		[17, 72, 74]	7
	Examine how community-wide campaigns can be institutionalized, such as through community coalitions.		[17]	2
	Systematically test implementation of multi-component physical activity interventions, including core components (e.g., sedentary time, sleep, nutrition) and implementation strategies.		[12, 14]	4

*An “x” in the health equity column denotes opportunity statements that mention social determinants of health or health disparities; reach, representativeness, or analysis by demographic items; intervention tailoring to improve reach; or other methods of reaching diverse priority populations

**Table 8:** Surveillance: opportunities (*n* = 9) by social ecological level, with health equity considerations

Social ecological level	Surveillance opportunities	Health equity*	Source references	Original statements (*n*)
Policy, systems, and environment	Conduct research on how to best incorporate social, environmental, and policy determinants of physical activity in surveillance systems.	x	[72, 77]	4
	Improve and institutionalize data collection to assess active transportation patterns, including by income, rurality, race/ethnicity, and socioeconomic status.	x	[17, 72]	5
	Compile and analyze national surveillance data to monitor walking and walkability.		[73]	3
	Develop, deploy, and test the effectiveness of systems to monitor physical education implementation and effectiveness.		[74]	2
	Expand surveillance systems to examine contributions of recreation, fitness, and park facilities to physical activity at community levels.		[72, 75]	2
	Update surveillance systems and constructs to prioritize transportation, workplace, and park sectors.		[72, 73, 75]	2
	Define and test a common framework to evaluate PSE implementation, effectiveness, and maintenance that can be integrated in existing surveillance systems.		[12, 72, 73, 75]	7
Crosscutting	Increase the use of valid, objective measures (e.g., device-based) versus reported physical activity measures in research and surveillance.		[12, 17, 47, 60, 72, 73, 75, 77, 78]	17
	Systematically assess physical activity behaviors of children and adolescents during school.		[72, 74]	2

*An “x” in the health equity column denotes opportunity statements that mention social determinants of health or health disparities; reach, representativeness, or analysis by demographic items; intervention tailoring to improve reach; or other methods of reaching diverse priority populations

## Discussion

A qualitative document analysis combined with expert consultation was used to organize physical activity research opportunities into a framework structured according to translational research phase and social ecological level. The resultant framework reveals opportunities for funders, researchers, policymakers, and practitioners to strategically advance their work, with specific attention to improving health equity. The framework could also facilitate communication among scientific sectors about resources, opportunities, priorities, and needs. In addition, it could be used as a resource to assist funders (e.g., CDC and NIH) with organizing funding repositories (e.g., the Office of Disease Prevention repository [79] could align with the PARO framework). The opportunities included in the framework also highlight gaps in current evidence throughout the process of translating physical activity research into practice. The framework provides prospects for designing efficacy and effectiveness trials with an eye toward dissemination and implementation, developing strategies for improving dissemination and implementation, and using community- and practitioner-engaged approaches across translational research science phases. The framework is intended to synthesize opportunities in consensus documents, reports, guidelines, and other sources, not state the official positions of federal agencies. It does not represent an articulation of member agencies’ priorities in their annual strategic plans.

First, most opportunity statements (76%) did not include health equity considerations. This may reflect the state of the field during the time the sources were published, as calls for health equity in physical activity have proliferated since 2020 [70, 80–82]. With these recent calls to action, there are opportunities to address health equity through better integrating community- and practitioner-engaged approaches to tailor interventions and improve reach. Two such approaches are Community-Based Participatory Research (CBPR) [83] and Integrated Research-Practice Partnerships (IRPPs) [84]. CBPR equitably involves community members and researchers in all aspects of the research process [83], while IRPPs include and equally value researcher and practitioner input [84]. Both approaches aim to bridge the gap between knowledge and practice, ensuring that research findings are pertinent to community and organizational needs and readily translatable into action [84, 85]. Interventions developed and tested using CBPR and IRPP approaches are more likely to be adopted, reach priority populations, and be sustained long-term [84–87]. To advance the scientific understanding of efficacious interventions and promote health equity, it is recommended that research agendas actively encourage early-phase studies that incorporate CBPR or IRPP approaches [88]. By doing so, researchers can ensure that interventions developed are grounded in a deep understanding of community needs, effective, and designed for broad dissemination and implementation [89, 90].

Second, more of the opportunity statements were related to interventions at the PSE level than individual and interpersonal levels. This aligns with recent evidence and funding areas and the recognition that supportive physical activity policies and environments must exist for individual-level behavior change efforts to succeed [17, 40–42, 91]. However, PSE interventions are difficult to evaluate because of complex research designs that can require collaboration between researchers, policymakers, and professionals from multiple sectors, such as public health, transportation, urban planning, and parks and recreation [1, 92]. Increased capacity, resources, and cross-sector collaboration could improve PSE implementation and evaluation [93]. The framework developed and reported in this manuscript could aid in these collaborative efforts, as it suggests the need for researchers to collaborate with policymakers and practitioners to advance physical activity research.

For example, one opportunity statement at the PSE level is “Investigate the effectiveness and maintenance of point-of-decision prompts, including periodic boosters.” Point-of-decision prompts (posters that encourage using stairs instead of elevator) can be challenging for community-based organizations to evaluate with limited time and resources, as typical evaluation requires days of pre- and post-intervention observation that may include repeated follow-up observations at periodic timepoints [94]. Partnerships between researchers (e.g., through student projects) and intervention sites (e.g., organizations that use posters and are interested in improving employee health) could enhance data collection options [94].

Another example that highlights the need for multi-sector collaboration is “Develop conceptual frameworks and systemic measures to improve social determinants of health in promoting physical activity.” Funders could provide support to researchers, who could engage with practitioners to develop a pragmatic framework and measures. For example, this could be accomplished through a request for proposals issued to conceptualize and measure structural determinants of health to supplement existing County Health Rankings & Roadmaps data (including physical activity access and inactivity measures) [95]. Multi-sector collaborations among health, urban planning, transportation, parks and recreation sectors, for example, could be encouraged to strengthen proposals.

**Fig. 1:**
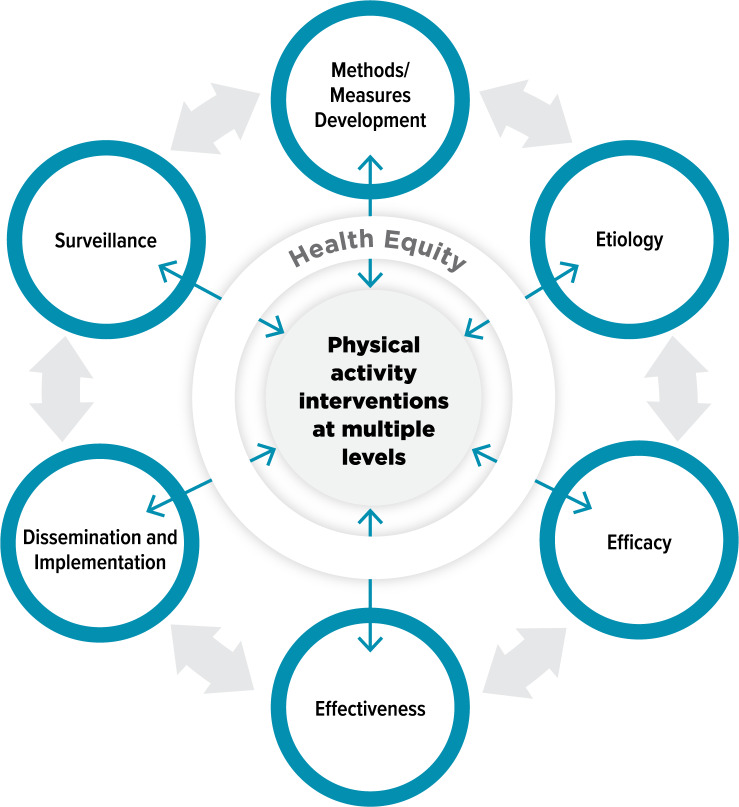
Translational research model of physical activity research

Next, related to translational research phases, although intervention development was included in the coding guide as a translational research phase (as it is specifically included in public health planning models as a step preceding intervention testing) [36, 63–65], no condensed opportunity statements were coded as intervention development. This is unsurprising, as a multitude of evidence-based interventions exist (and are reflected in the diverse interventions included in the framework tables) [17]. Efforts are needed to (1) tailor these interventions for priority populations to reduce physical activity disparities and improve health equity and (2) scale them to reach additional populations and settings. However, this requires adopting and adapting existing evidence-based interventions versus focusing only on developing new interventions [96–98]. Thus, Fig. 1 was developed to revise the translational research science model proposed at the onset of the study and better capture the translational research pipeline needed to advance physical activity research. In this model, there is a need for physical activity interventions at multiple social ecological levels, which are informed by opportunities for methods/measures development, etiology, efficacy, effectiveness, dissemination and implementation, and surveillance research through a bidirectional relationship. The translational research phases exist in an iterative process, with each phase building evidence and leading to subsequent (or, at times, previous) phases to understand, test, and measure physical activity promotion efforts.

Relatedly, most condensed opportunity statements pertained to effectiveness research and dissemination and implementation research, with few focusing on efficacy or etiology research. Given the previously noted trends in physical activity research [1, 36, 37], this is an important finding that could encourage researchers to conduct more studies that test interventions and efforts to scale them. Of note, related to dissemination and implementation, there are opportunities for collaborations between researchers and practitioners to improve health equity through building practice-based evidence to address the needs of diverse communities, which are also often historically and intentionally excluded [22]. One illustrative example is the opportunity statement “Study the effects of increased access to parks and facilities in communities with less access and in populations experiencing health disparities.” Researchers and practitioners could work together to evaluate and understand factors that lead to greater access to parks in communities with less access and collaboratively develop implementation strategies to increase access [99, 100]. Understanding these factors could influence local leaders and community members to support the development of parks in areas with lower access in a way that discourages displacement and gentrification [101]. Overall, while the PARO framework details research opportunities, it may also be useful to practitioners working to integrate evidence-based interventions, collaborate with researchers, and inform practice-based research [102].

While fewer statements focused on efficacy or etiology research, there is still a need for these studies to build evidence for effectiveness and dissemination and implementation studies. Etiological research can aid in understanding the complex variables influencing physical activity, including genetics, environment, cultural norms, socioeconomic status, and personal beliefs that influence a person’s desire and ability to engage in physical activity [43, 76]. For example, related to the opportunity statement “Identify populations at risk for physical inactivity and conduct research to understand their specific barriers,” research may investigate the influence of urban planning on the probability of using walking or biking as a means of transportation or how job and family responsibilities hinder or promote regular physical activity [103–105]. Next, related to efficacy, there is a need for early phase studies to progress to effectiveness and dissemination and implementation research. These trials can be designed to include external as well as internal validity considerations by recruiting more diverse populations or collecting data on implementation outcomes (e.g., adoption, implementation, maintenance) [29, 35, 106]. For example, the opportunity statement “Build evidence of efficacy for primary care physical activity interventions” could be enhanced by incorporating a multi-level contextual study (e.g., with administrators, health care providers, and patients) to understand implementation barriers and facilitators and inform the selection of relevant implementation strategies [31]. Doing so can lead to interventions that are more likely to work in their intended real-world settings and benefit populations experiencing health disparities [31, 107, 108].

### Limitations

This study was not a systematic (or other structured) review. Instead, the research team relied on the content area experts’ knowledge of current physical activity promotion efforts and workgroups to select sources from the government, intergovernmental, or non-profit organizations, which may not necessarily represent all research opportunities in the field. As well, the number of original opportunity statements condensed into each condensed opportunity statement should be interpreted with caution. That is, a higher frequency of original opportunity statements may reflect the fact that more interest exists in specific areas (e.g., there are multiple reports specific to youth physical activity) rather than imply that there is a greater need for research.

One additional limitation is that it was difficult to determine the translational research phase of multifaceted opportunity statements. For example, “Assess reliability and validity of surveillance questions” was coded as methods/measures development but is also related to surveillance. Efficacy versus effectiveness research opportunity statements were also difficult to discern within the sometimes limited details provided; flexibility should be used when interpreting these statements. Multiple study designs exist to answer research questions, and researchers can use their best judgment and recommendations from the field when determining whether effectiveness or efficacy studies are needed [108, 109]. Users should consider balancing rigor, relevance, and rapidity to speed the translation of research to practice and achieve real-world benefits [110]. Physical activity priorities will likely change over time, and the PARO framework will need to be updated as new research opportunities are identified.

## Conclusions

The PARO framework developed by the authors organizes physical activity research opportunities from government, intergovernmental, and non-profit organization sources and assists researchers and other groups in prioritizing opportunity areas and topics more efficiently. Overall, the opportunities noted here are consistent with physical activity researchers’ calls for more effectiveness research, dissemination and implementation research, and studies addressing PSE-level interventions. Furthermore, additional evidence is needed to enhance our ability to achieve health equity for all and ensure health benefits reach disinvested communities and populations experiencing health disparities. Ultimately, the PARO framework can serve as a tool for advancing the physical activity research agenda, informing future guidelines (e.g., the next version of the Physical Activity Guidelines for Americans), and building both evidence-based practice and practice-based evidence.

## Supplementary Information

Supplementary Material 1.

Supplementary Material 2.

Supplementary Material 3.

## Data Availability

All data generated or analyzed during this study are included in this published article and its supplementary information files. Coding and reconciliation audit trail files are available from the corresponding author on reasonable request.

## Abbreviations

CBPR: Community-Based Participatory Research
CPSTF: Community Preventative Services Task Force
IRPP: Integrated Research-Practice Partnerships
NCCOR: National Collaborative on Childhood Obesity Research
PARO: Physical Activity Research Opportunities
PSE: Policy, Systems, or Environment
