# Supplementary material for: Development of the Physical Activity Research Opportunities (PARO) framework
Source: Int J Behav Nutr Phys Act. 2025 Nov 26;22:152. doi: 10.1186/s12966-025-01843-3 (PMC12659305; doi:10.1186/s12966-025-01843-3)
Supplement: Supplementary file 3 — Supplementary Material 3. [file 12966_2025_1843_MOESM3_ESM.docx]

**Crosstabulation of combined physical activity research recommendations (N=87) by translational phase and intervention level**

|  | | Translational phase  n (%) | | | | | | Total recommendations by intervention level  n (%) |
| --- | --- | --- | --- | --- | --- | --- | --- | --- |
|  |  | Methods and measures development | Etiology | Efficacy | Effectiveness | Dissemination and implementation | Surveillance |  |
| Intervention Level  n (%) | Individual and interpersonal | 1 (1) | 1 (1) | 4 (5) | 17 (20) | 3 (3) | 0 (0) | **26 (30)** |
|  | Policy, systems, and environment | 4 (5) | 3 (3) | 0 (0) | 14 (16) | 5 (6) | 7 (8) | **33 (38)** |
|  | Crosscutting | 3 (3) | 2 (2) | 2 (2) | 13 (15) | 6 (7) | 2 (2) | **28 (32)** |
| Total recommendations by translational phase  n (%) | | **8 (9)** | **6 (7)** | **6 (7)** | **44 (51)** | **14 (16)** | **9 (10)** | **87 (100)** |
